# Experience with subcutaneous immunoglobulin therapy in two pediatric cases of immune thrombocytopenia purpura

**DOI:** 10.1186/1710-1492-8-S1-A23

**Published:** 2012-11-02

**Authors:** H Chapdelaine, H Decaluwe, MC Levasseur, F De Deist, E Haddad

**Affiliations:** 1 Department of Pediatrics, CHU Sainte-Justine, Montréal, Québec, Canada, H3T 1C5; 2 Department of Microbiology and Immunology, Université de Montréal, Montréal, Québec, Canada, H3T 1J4; 3 Department of Microbiology and Immunology, CHU Sainte-Justine, Montréal, Québec, Canada, H3T 1C5

## Background

Immune thrombocytopenia purpura (ITP) can co-exist with primary immunodeficiencies. Intravenous immunoglobulin (IGIV) therapy is an effective treatment. Subcutaneous immunoglobulin (SCIG) formulations that can be home-delivered have recently been developed. We describe 2 cases of pediatric ITP associated with hypogammaglobulinemia, treated with SCIG.

## Case description

### Case 1

A 14-year old male presented with a symptomatic thrombopenia. Infusions of IGIV led to an immediate improvement in platelet count. However, he experienced post-infusion intractable headaches, nausea and vomiting, which recurred after subsequent infusions. Intravenous anti-D therapy resulted in a severe allergic reaction. Short course prednisone protocol was implemented for symptomatic episodes. Preliminary blood work for splenectomy revealed low IgG level. The patient was put on SCIG replacement therapy (116 mg/kg/week). He experienced only one relapse since, which remained corticoresponsive.

### Case 2

A 14-year-old male was referred for asymptomatic thrombo-neutropenia. Extensive work-up was normal. He was administered IVIG after which the platelet count quickly normalized. Eighteen months later, he developed monoarthritis and generalized adenopathies. Dominant lymph node biopsy showed reactive lymphoid hyperplasia. The patient started to experience recurrent thrombopenia flare-ups, needing monthly IVIG. Laboratory results indicated low IgM and IgG levels. To prevent further episodes, he received prophylactic monthly IVIG for 6 months before switching to SCIG (135 mg/kg/week). Platelet and also neutrophil levels normalized, which was not achieved with IGIV.

In both patients, SCIG therapy was well-tolerated with no adverse events occurring.

## Conclusion

These results suggest that SCIG can be an effective and convenient treatment of pediatric ITP.

